# The Role of IL-17 and TH17 Cells in the Bone Catabolic Activity of PTH

**DOI:** 10.3389/fimmu.2016.00057

**Published:** 2016-02-17

**Authors:** Roberto Pacifici

**Affiliations:** ^1^Division of Endocrinology, Metabolism and Lipids, Department of Medicine, Emory University, Atlanta, GA, USA; ^2^Immunology and Molecular Pathogenesis Program, Emory University, Atlanta, GA, USA

**Keywords:** T cells, PTH, IL-17, osteoblasts, osteocytes, bone

## Abstract

Osteoimmunology is field of research dedicated to the study of the interactions between the immune system and bone. Among the cells of the immune system that regulate the skeleton in health and disease are T lymphocytes, T cells secrete inflammatory/osteoclastogenic cytokines such as RANKL, TNF, and IL-17, as well as factors that stimulate bone formation, including Wnt ligands. In addition, T cells regulate the differentiation and life span of stromal cells via CD40L and other costimulatory molecules expressed on their surface. Consensus exists that parathyroid hormone (PTH) induces bone loss by increasing the production of RANKL by osteocytes and osteoblast. However, new evidence suggests that PTH expands Th17 cells and increases IL-17 levels in mice and humans. Studies in the mouse of further shown that Th17 cell produced IL-17 acts as an “upstream cytokine” that increases the sensitivity of osteoblasts and osteocytes to PTH. As a result, PTH stimulates osteocytic and osteoblastic release of RANKL. Therefore, PTH cause bone loss only in the presence of IL-17 signaling. This article reviews the evidence that the effects of PTH are mediated not only by osteoblasts and osteocytes, but also T cells and IL-17.

## Introduction

Parathyroid hormone (PTH) is an important regulator of calcium and phosphorus concentrations in extracellular fluid. Physiologic levels of circulating PTH are essential for maintaining serum and urinary calcium levels within their normal range. Chronic excessive PTH production is a cause of skeletal and extra skeletal disease. Secondary hyperparathyroidism has been implicated in the pathogenesis of senile osteoporosis (1), while primary hyperparathyroidism (PHPT) is associated with accelerated bone loss (2) and osteoporosis (3–5).

Primary and secondary hyperparathyroidism are mimicked by continuous PTH (cPTH) infusion. cPTH and PHPT increase bone turnover in trabecular and cortical bone, as evidenced by elevations in histomorphometric and biochemical markers of resorption and formation (6–8), whereas PHPT and cPTH treatment cause cortical bone loss by enhancing endosteal resorption through stimulation of osteoclast formation, activity, and life span (3, 8, 9). Severe chronic elevations of PTH levels may also lead to trabecular bone loss (3, 8), although PHPT and cPTH treatment often induce a modest increase in cancellous bone (4–6, 10).

The effects of cPTH on bone result from its binding to the PTH/PTH-related protein (PTHrP) receptor (PPR or PTHR1), which is expressed not only on BM stromal cells (SCs), osteoblasts, and osteocytes (11, 12) but also on T cells (13) and macrophages (14). SCs and osteoblasts were the first targets of PTH to be identified, and earlier consensus developed that the catabolic effect of cPTH is mostly mediated by enhanced production of RANKL and decreased production of OPG by SCs and osteoblasts (15–17). More recent studies in mice with deletion and/or overexpression of PPR and RANKL in osteocytes (12, 18–20) lead to the recognition that osteocytes represent essential targets of PTH in bone, and that increased production of RANKL by osteocytes plays an important role in cPTH-induced bone loss (12, 19). However, some reports have ascribed a key role to OB produced RANKL (21). Moreover, studies have also shown that PPR signaling in T cells stimulates the release of TNF (22), and that deletion of T cells, T cell production of TNF, or PPR signaling in T cells prevents cPTH-induced bone loss (22, 23), as effectively as deletion of PPR signaling in osteocytes. Because of these reports, T cells are now recognized as a second critical target of PTH in bone. Controversy remains on the relative relevance of T cells, osteocytes, and osteoblasts for the activity of PTH. However, new evidence suggests that PTH expands Th17 cells and increases IL-17 levels in mice and humans (24). Studies in the mouse of further shown that Th17 cell-produced IL-17 acts as an “upstream cytokine” that increases the sensitivity of osteoblasts and osteocytes to PTH. As a result, PTH stimulates osteocytic and osteoblastic release of RANKL, and thus cause bone loss, only in the presence of intact IL-17 signaling. This article focuses on the role of Th17 cell-produced IL-17 in the mechanism of action of PTH in bone.

## TH17 Cells and PTH-Induced Bone Loss

The discovery that T lymphocytes express functional PPR (13) and respond to PTH (25) prompted investigations on the role of T cells as mediators of the effects of cPTH in bone. Early studies revealed that levels of PTH typically found in PHPT require the presence of T cells to induce bone loss (26, 27), whereas conditions that cause extreme elevations in PTH levels induce bone loss via T cell-independent mechanisms (28–31).

T cells exert complex activities that are relevant for the effects of PTH in bone, including stimulating the production of TNF by both CD4^+^ and CD8^+^ T cells (22). Since CD8^+^ cells are more abundant in the BM than CD4^+^ cells, most of the TNF produced in the BM in response to cPTH originates from CD8^+^ cells (22). TNF stimulates osteoclast formation and activity via multiple mechanisms, which include increased production of RANKL by all osteoblastic cells including osteocytes. Attesting to the relevance of T cell produced TNF, cPTH fails to induce bone loss and stimulate bone resorption in mice specifically lacking T cell TNF production (22). PTH induces T cell production of TNF via direct activation of PPR signaling in T cells (22). Conditional silencing of the PTH receptor PPR in T cells blunts the stimulation of bone resorption induced by cPTH without affecting bone formation, thus blocking cortical bone loss and converting the effects of cPTH in trabecular bone from catabolic to anabolic (22). These findings demonstrate the critical relevance of direct PPR signaling in T cells.

cPTH stimulates bone cells and immune cells to release growth factors and cytokines. Among them are TGFβ, IL-6, and TNF (22, 32–34). TGFβ and IL-6 direct the differentiation of naive CD4^+^ cells into Th17 cells (35–37).

Th17 cells are the most osteoclastogenic subsets of T cells (38). Th17 cells are defined by their capacity to produce the cytokine IL-17. Th17 cells are constitutively present at mucosal surfaces, especially in the intestinal lamina propria (39). Th17 cells play a pivotal role in the bone loss of inflammatory conditions such as psoriasis, rheumatoid arthritis, periodontal disease, and IBD (40, 41). Th17 cells potently induce osteoclastogenesis by secreting IL-17, RANKL, TNF, IL-1, and IL-6, along with low levels of IFNγ (42–44). IL-17 stimulates the release of RANKL by osteoblasts and osteocytes (24, 38) and potentiates the osteoclastogenic activity of RANKL by upregulating RANK (45). IL-17 provides an important connection between T cells and osteocytes as this T cell cytokine regulates osteocytic RANKL production (24), which is one key effect of PTH on osteocytes (12, 19).

Studies with agents neutralizing TNF have implicated TNF in the generation of Th17 cells in rodents and humans (46–48). Moreover, PTH binding to PPR activates the G protein-coupled receptor subunit GαS, leading to the generation of cAMP (49). Accumulation of cAMP in CD4^+^ cells and the resulting Ca^2+^ influx further promote Th17 cell differentiation and activity (50). Therefore, cPTH could expand Th17 cells via several mechanisms. This reasoning prompted investigations on the relationship between cPTH treatment and Th-17. Murine studies have revealed that cPTH treatment increases the relative and absolute frequency of Th17^+^ cells and the levels of IL-17 in peripheral blood, spleen, and BM (24). Detailed analysis of samples of peripheral blood revealed that cPTH increased IL-17 levels in purified peripheral blood CD4^+^ cells and unfractionated peripheral blood nucleated cells, but not in CD4^+^ cell-depleted peripheral blood nucleated cells, indicating that CD4^+^ cells represent the major source of IL-17 mRNA in peripheral blood cells. Moreover, cPTH also increases the mRNA levels of the Th17-inducing transcription factors RORα and RORγt in peripheral blood, spleen, and BM CD4^+^ T cells. This effect of cPTH is specific for Th17 cells because cPTH does not expand murine Th1 cells, Th2 cells, and regulatory T cells.

Mechanistic studies have disclosed that cPTH increases Th17 cell differentiation in the BM and the spleen. By contrast, cPTH does not stimulate Th17 cell proliferation (24). Surprisingly, these studies have shown that cPTH expands Th17 cells and increases the production of IL-17 via TNF, and more specifically, the pool of TNF produced by conventional CD4^+^ and CD8^+^ T cells. This conclusion is based on the fact that cPTH failed to expand BM and splenic Th17 calls in TNF-null mice and in T cell-null mice previously subjected to adoptive transfer of TNF^−/−^ T cells. The latter experimental model is particularly significant, because the host mice possess all physiologic sources of TNF except for T cells. Yet, specific ablation of T cell produced TNF is sufficient to block the capacity of cPTH to expand Th17 cells, increase BM IL-17 levels and prevent bone loss (24). Additional T cell reconstitution studies revealed that TNF directly targets Th17 precursors. To reach this conclusions, investigators reconstituted T cell-null mice with CD4^+^ cells from TNFR1^−/−^ and TNFR2^−/−^ mice and then treated the host mice with cPTH. Under these conditions, cPTH expanded BM Th17 cells and increase BM IL-17 levels in mice with TNFR2^−/−^ T cells but not in those with TNFR1^−/−^ T cells, thus demonstrating that TNFR1 signaling is required for cPTH to induce the differentiation of CD4^+^ cells into Th17 cells. In addition to IL-6 TGFβ and TNF, several cytokines are known to promote Th17 cell expansion. Among them are the T cell produced factor IL-21 and the macrophage/dendritic cell produced cytokine IL-23. cPTH treatment increases the BM levels of IL-21, and IL-23R in WT mice. By contrast, cPTH did not increase the levels of these cytokines in TNF^−/−^ mice. Thus, IL-21 and IL-23 are likely to contribute to expansion of Th17 cells induced by cPTH. However, cPTH upregulates these factors via TNF.

As stated above, PTH receptor signaling activates GαS, leading to the generation of cAMP (49), which further promotes TH17 cell differentiation via Ca^2+^ influx (50). One mechanism by which activation of GαS in CD4^+^ cells could promote Th17 cell differentiation is increased sensitivity to TNF. To investigate this hypothesis, investigators used GαS^ΔCD4,8^ mice, a strain characterized by silenced GαS signaling in CD4^+^ and CD8^+^ T cells. Pivotal experiments revealed that cultures of CD4^+^ cells from cPTH-treated control mice yielded a higher number of Th17 cells as compared to those from vehicle-treated mice (24). By contrast, cultures of CD4^+^ cells from vehicle and cPTH-treated GαS^ΔCD4,8^ mice yielded similar numbers of Th17 cells, demonstrating that cPTH increases the sensitivity of nascent Th17 cells to TNF via GαS signaling in CD4^+^ cells. Attesting to the relevance of GαS signaling in CD4^+^ cells for Th17 cell generation, cPTH was found not to expand BM and splenic Th17 cells and not to exerts its bone catabolic activity in GαS^ΔCD4,8^ mice, demonstrate that silencing of Gαs in T cells prevents the expansion of Th17 cells and the bone loss induced by cPTH (24).

Signaling events downstream of GαS include cAMP generation (49) and activation of L-type calcium channels (51), which promote Th1 and Th17 cell differentiation (50). This evidence suggests the possibility that treatment with the L-type calcium channel blocker diltiazem may blunt the differentiation of CD4^+^ cells into Th17 cells (50) and prevent the bone loss induced by cPTH.

This hypothesis was tested in murine studies that revealed that diltiazem blocks the expansion of Th17 cells, the increase in bone resorption, and the loss of cortical and trabecular bone induced by cPTH. The finding may suggest a potential therapeutic role for L-type calcium channel blockers in the treatment of hyperparathyroidism.

## Neutralization of IL-17A or Silencing of IL-17RA Block cPTH-Induced Bone Loss

The finding that cPTH increases the levels of IL-17 does not demonstrate that IL-17 plays a role in the bone catabolic activity of cPTH. To demonstrate the relevance of IL-17 in the mechanism by which cPTH alters skeletal homeostasis mice were treated with cPTH and a neutralizing antibody directed against murine IL-17 (IL-17 Ab). These studies revealed that IL-17 Ab completely prevents the loss of cortical and trabecular bone induced by cPTH. Analysis of biochemical and histomorphometric indices of bone turnover revealed that neutralization of IL-17 blunts the bone catabolic activity of cPTH by decreasing bone resorption (24).

To confirm these findings, additional experiments were conducted utilizing IL-17RA-null mice, a strain lacking the heterodimeric receptor IL-17RA/IL-17RC known as IL-17RA (52, 53). IL-17 signaling is silenced in IL-17RA^−/−^ mice (54). These studies disclosed that cPTH stimulates bone resorption and indices bone loss in control mice but not in IL-17RA mice (24), thus demonstrating that silencing of IL-17 signaling prevents the bone catabolic activity of cPTH.

## IL-17 is an Upstream Cytokine Required for cPTH to Increase Rankl Release by Osteoblasts and Osteocytes

**Figure 1:**
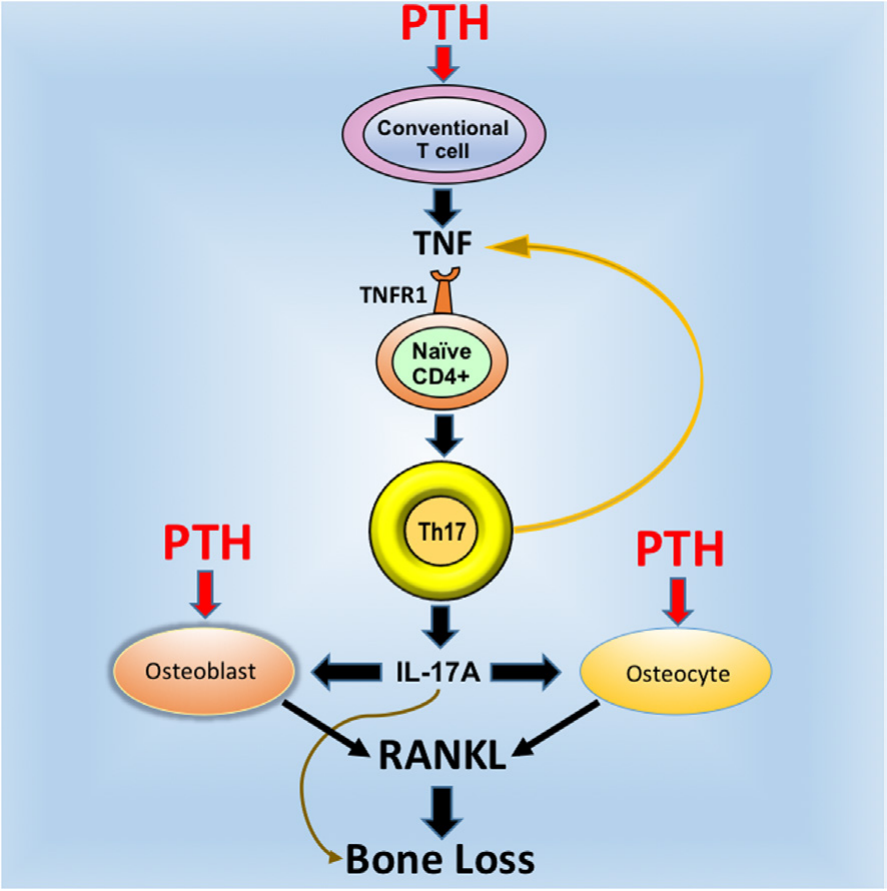
**Schematic representation of the mechanism of action of cPTH in bone**. PTH binds to the PTH receptor PPR expressed in conventional CD4^+^ and Cd8^+^ T cells and induces the secretion of TNF. This cytokines induces the differentiation of naive CD4^+^ cells into Th17 cells via TNFR1 signaling. Th17 cells release additional TNF, which further stimulates Th17 differentiation. More importantly, Th17 cells secrete IL-17, which targets osteocytes and osteoblasts, thus increasing their sensitivity to TNF. In the presence of IL-17, PPR activation in osteocytes and osteoblasts stimulates these cells to release RANKL, which stimulates bone resorption and induces bone loss. Silencing of IL-17 or IL-17RA signaling blocks the capacity of cPTH to stimulate the production of RANKL by osteocytes and osteoblasts. Reproduced with permission from Ref. (24).

Osteocytes and the pool of RANKL produced by osteocytes are crucial for the activity of cPTH (12, 19, 20). On the other hand, studies form our laboratory have shown T cells are an additional important target of PTH (55). The fact that silencing of PPR signaling in T cells and osteocytes induces similar bone sparing effects is in keeping with a “serial circuit” regulatory model, where signals from one population affect the response to cPTH of the other. Since T cells and osteocytes have limited physical contacts, the cross talk between these populations is likely mediated by a soluble factor. IL-17A is a probable candidate because it is a potent inducer of RANKL in organ cultures containing osteoblasts and osteocytes (56). In support of this hypothesis, investigations have shown that neutralization of IL-17 via treatment with IL-17 Ab and deletion of IL-17RA block the capacity of cPTH to increase the production of RANKL by osteocytes and osteoblasts (24). These data indicate that IL-17A increases the sensitivity of osteoblasts and osteocytes to cPTH, thus enabling these lineages to release RANKL when stimulated by cPTH. Therefore, IL-17 mediates the bone catabolic activity of cPTH by upregulating the production of RANKL by osteocytes and osteoblasts (Figure 1).

It is important to underscore that the available published data suggest that T cells, osteoblasts, and osteocytes are all required for cPTH and by extension, PHPT, to induce bone loss. By contrast, osteocytes, but not T cells and IL-17, are required for physiologic levels of endogenous PTH to regulate bone remodeling. In fact, mice lacking PPR signaling in osteocytes have high baseline bone volume (12), while IL-17RA-null mice and those lacking PPR signaling in T cells (22, 57) have a normal bone volume.

## Increased Production of IL-17A in Humans Affected by Primary Hyperparathyroidism

While numerous studies have investigated the role of immune cells and cytokines in the mechanism of action of PTH in animal models, little information is available in humans.

**Figure 2:**
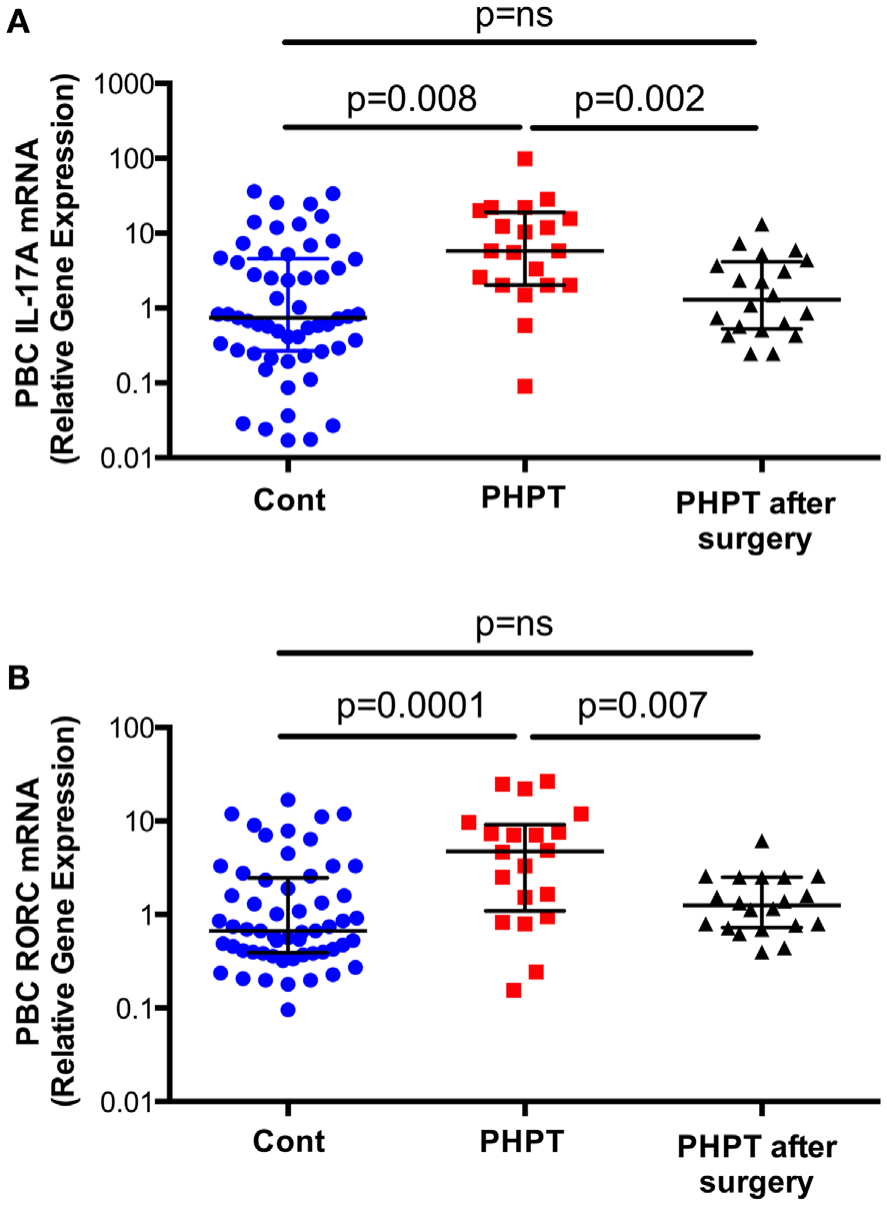
**Levels (Median ± interquartile range) of IL-17A (Panel A) and RORC mRNAs (Panel B) in healthy controls (*n* = 57) and subjects with PHPT before (*n* = 20) and after parathyroidectomy (*n* = 20)**. Data were analyzed by Mann–Whitney (healthy controls vs. PHPT before surgery and healthy controls vs. PHPT after surgery) and Wilcoxon matched pairs signed rank tests (PHPT vs. PHPT after surgery) as the data were not normally distributed according to the Shapiro–Wilk normality test. Reproduced with permission from Ref. (24).

To investigate the effects of PHPT on the production of cytokines, unfractionated peripheral blood nucleated cells were obtained from 57 healthy controls and 20 similar subjects affected by PHPT. In PHPT patient’s blood samples were obtained before surgery and 1 month after successful resolution of PHPT by parathyroidectomy. This study revealed (Figure 2) that the mRNA levels of IL-17A in unfractionated peripheral blood nucleated cells were approximately threefold higher in PHPT patients than in healthy controls (24). Moreover, surgical restoration of normal parathyroid function was associated with the normalization of IL-17A levels. Furthermore, the mRNA levels of the IL-17-inducing transcription factor RORC were approximately threefold higher in PHPT patients before surgery than in healthy controls and parathyroidectomy was followed by a decrease in RORC mRNA levels. PTH levels were directly correlated with IL-17A level and the differences in IL-17A and RORC levels between healthy controls and PHPT patients remained significant even after adjustment for age and gender by a multiple regression model. These findings suggest that increased IL-17A gene expression in PHPT patients is due to increased levels of circulating PTH.

## Conclusion

Remarkable progress has been made in understanding how T cells participate in the regulation of bone remodeling in health and disease. An impressive amount of work published in the last 10 years has led to the recognition that T cells play an unexpected role in the regulation of bone resorption and bone formation through a variety of mechanisms and the involvement of specialized cell lineages such as Th17 cells and Tregs. Work remains to be done to fully understand the cross-talk between bone cells and immune cells.

Some confirmation of the relevance of T cells in human bone diseases has now been reported but much remains to be done. Most of the human evidence has been accrued in studies on the pathogenesis of postmenopausal osteoporosis. For example, evidence begins to emerge in favor of a role of T cell produced TNF in postmenopausal bone loss in women (58, 59) and that in humans estrogen deficiency expands RANKL-expressing T cells and B cells (60, 61). Moreover, a role for IL-1 and TNF in humans is supported by reports that menopause increases the levels of these factors (62–66), while treatment with inhibitors of IL-1 and TNF prevents the increase in bone resorption induced by estrogen deficiency (67). A recent report from our laboratory show that PHPT increases IL-17 production in humans, an abnormality which is resolved by successful parathyroidectomy. Our studies show that in mice, the bone loss induced by cPTH is prevented by the calcium channel blocker diltiazem, and IL-17 Ab. Direct clinical applications of these finding arise because L-type calcium channel blocker are available, while anti-human IL-17 Abs and IL-17 receptor Abs are under investigation as therapeutic agents in psoriasis and spondyloarthropathy (68–71).

## Author Contributions

The author confirms being the sole contributor of this work and approved it for publication.

## Conflict of Interest Statement

The author declares that the research was conducted in the absence of any commercial or financial relationships that could be construed as a potential conflict of interest.
